# Collaborative Settings Increase Dishonesty

**DOI:** 10.3389/fpsyg.2021.650032

**Published:** 2021-05-14

**Authors:** Youhong Du, Weina Ma, Qingzhou Sun, Liyang Sai

**Affiliations:** ^1^Center for Cognition and Brain Disorders, The Affiliated Hospital of Hangzhou Normal University, Hangzhou, China; ^2^Institute of Psychological Science, Hangzhou Normal University, Hangzhou, China; ^3^Zhejiang Key Laboratory for Research in Assessment of Cognitive Impairments, Hangzhou, China; ^4^Department of Education, Institute of Psychological Sciences, Hangzhou Normal University, Hangzhou, China; ^5^School of Management, Zhejiang University of Technology, Hangzhou, China

**Keywords:** collaborative settings, dishonesty, die-rolling task, cooperation, deception

## Abstract

The present study examines whether collaborative situations make individuals more dishonest in face-to-face settings. It also considers how this dishonesty unfolds over time. To address these questions, we employed a sequential dyadic die-rolling task in which two participants in a pair sitting face-to-face received a payoff only if both reported the same outcome when each one rolled their die. In each trial, one participant (role A) rolled a die first and reported the outcome. Then, the second participant (role B) was informed of A’s reported number, rolled a die as well, and reported the outcome. If their reported outcomes were identical, both of them received a reward. We also included an individual condition in which an individual subject rolled a die twice and received a reward if he/she reported the same die-roll outcome. We found that B lied significantly more than participants in the individual condition, whereas A lied as much as participants in the individual condition. Furthermore, when collaborating, more and more participants (both A and B) became dishonest as the game progressed, whereas there was no such trend among participants in the individual condition. These findings provide evidence indicating that collaborative settings increase dishonesty and that this effect becomes more evident as the collaboration progress.

## Introduction

Cooperation is essential to humans because it allows them to perform tasks more effectively and to develop trust (Kramer, 1999; Rempel et al., 2001). It also helps them to build relationships with one another (Bazerman et al., 2000; Kameda et al., 2005). For these reasons, individuals tend to prefer cooperation over working alone (Rand, 2017). However, in some situations, cooperation can involve violating certain moral rules. For example, corruption typically arises when people work together to obtain profits illegally (Gross et al., 2018). In a moral dilemma like this, will individuals be more inclined to cooperate and break moral rules or not to cooperate and obey them (e.g., honesty)?

Weisel and Shalvi (2015) were the first to examine the dishonesty of individuals in collaborative situations. They conducted an experiment involving sequential dyadic die-rolling, in which two participants were paid according to whether they reported the same number after rolling dice sequentially. Since the rolls were private, participants could misreport their actual outcomes. They found that the proportion of reported doubles was significantly higher than was to be expected if the participants had been honest. It was also higher than the number of doubles reported when individuals rolled and reported alone. These findings suggest that individuals are more dishonest in collaborative situations than they are in individual situations. Wouda and his colleagues replicated the experiments of Weisel and Shalvi (2015) and verified their findings (Wouda et al., 2017). Researchers argued that collaborative situations provide individuals with a good reason to justify their immoral behavior, leaving them more likely to be dishonest (Weisel and Shalvi, 2015; Soraperra et al., 2017).

Although the above findings suggest that collaborative situations increase dishonesty, it is worth noting that they ignored the fact that collaboration also typically involves increased observability and accountability. As such, reputational concerns may limit people’s willingness to break moral rules (Weisel and Shalvi, 2015). Weisel and Shalvi (2015), for example, asked their participants to sit in separate cubicles. The participants never met each other during the experiment, so the findings may be limited to such cases, where reputation plays a minor role. Therefore, it remains unknown whether individuals are as dishonest in face-to-face collaborative situations where they have concerns about their reputation. One primary aim of the current study is to address this issue.

Furthermore, while previous studies have demonstrated that individuals are more dishonest when collaborating with others, it is still unclear how this dishonesty unfolds over time. The previous evidence suggests that individuals are more likely to cooperate in multiple interactions because they may develop trust with each other over time (Levine and Schweitzer, 2015). In this case, it is expected that individuals will be more likely to become more dishonest as they collaborate over time. The previous research also suggests that individuals are likely to commit minor acts of unethical behavior but not major acts of unethical behavior because they can easily justify these minor acts. Furthermore, over time, individuals become more likely to engage in major forms of unethical behavior as it becomes easier for them to justify their conduct (Welsh et al., 2015). In this case, it is also expected that individuals will become more dishonest over time as they find it easier to justify their immoral behavior. The second aim of this study is to test whether individuals become more likely to collaborate through dishonesty. This will help us to gain a greater understanding of how dishonesty develops in collaborative situations and suggest ways of reducing it.

To address both of these research aims, the present study used a modified sequential dyadic die-rolling paradigm created by Weisel and Shalvi (2015). To increase observability and accountability, we asked participants to sit across from one another at a table. Also, a hidden camera was used to record the outcome of each die roll so that we could identify whether or not a participant lied in a specific trial by comparing the real outcome of each die roll with the outcome of a participant reported.

Based on the previous findings that have shown that collaboration can make it more likely that people will behave unethically (Weisel and Shalvi, 2015), we expected that participants would lie more when operating collaboratively than when operating alone. Moreover, since it becomes easier for participants to justify their immoral behavior over time (Welsh et al., 2015), and because the two participants would be able to develop trust with each other as the game progressed (Levine and Schweitzer, 2015), we expected that more participants would exhibit dishonest behavior over time as they collaborated more.

## Materials and Methods

### Participants

We conducted a prior power analysis using G*Power version 3.1.9.2. The parameters used in this calculation were alpha = 0.05 and power = 0.8. The effect size was derived from the previous study conducted by Weisel and Shalvi (2015). The analysis indicated that 53 participants would be needed for the collaborative condition and 27 participants would be needed for the individual condition. This would provide enough data to test the difference between them. Thus, 88 students who were not psychology majors were recruited from Hangzhou Normal University. Participants were paired with another person of the same gender with whom they were unacquainted. Participants were then randomly assigned to either the collaborative condition or the individual condition. One participant in the individual condition was excluded because the camera was broken and could not record data completely. This left a final sample of 30 dyads (three male dyads, *M* = 20.2 years; *SD* = 2.02) in the collaborative condition and 27 participants (one male, *M* = 19.74 years; *SD* = 1.58) in the individual condition. Written informed consent was obtained from each participant in accordance with the Declaration of Helsinki. The study was approved by the Ethics Committee of the Center for Cognition and Brain Disorder at Hangzhou Normal University.

### Experimental Procedure

**Figure 1:**
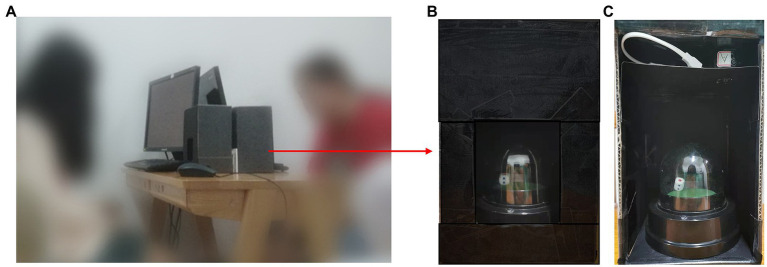
Experimental environment and materials. (A) The experimental environment. (B) The appearance of the automatic rolling dice device. (C) The internal structure of the automatic rolling dice device.

A pair of participants came into the laboratory and were randomly assigned to the collaborative condition or the individual condition. Participants in the collaborative condition were then randomly assigned to role A (A) or role B (B). In the individual condition, the same person acted in both roles. In both conditions, the pair of participants were sat across from each other and each had a computer screen and a device for rolling a die (Figure 1A).

**Figure 2:**
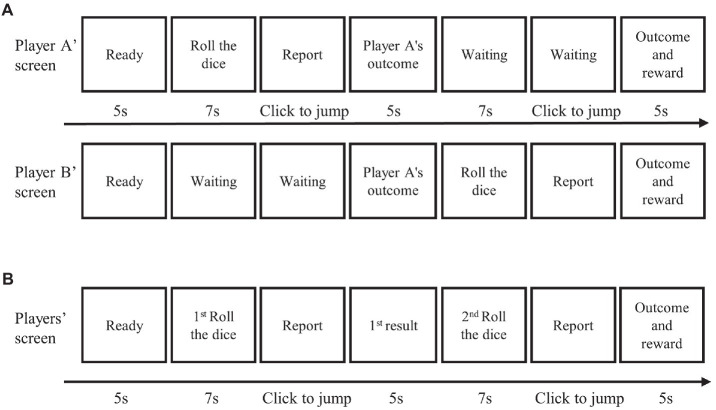
Experimental procedure. **(A)** The procedure in the collaborative condition. **(B)** The procedure in the individual condition.

Participants were told to play the dice-rolling game. While the experiment was going on, the participants were not allowed to talk to each other. In the collaborative condition, A rolled first and reported the outcome by typing a number on the computer. B was then informed of A’s outcome. Finally, B rolled and reported their outcome in turn. If their reported outcomes were identical, they both received a reward (see Figure 2A). The amount paid was the equivalent in RMB to the number reported on the dice. For example, if both A and B reported a roll of two, they would each earn ¥0.2; if both A and B reported six, they would each earn ¥0.6. In contrast to the previous studies, the participants were given an electronic rolling device so they could roll their die simply by pressing a button. They could then observe the die through a small window, which was only visible to them alone (Figure 1B). There was also an electronic light inside the box to allow participants to see the result of each roll clearly. However, unbeknownst to the participants, a mini camera was hidden at the top of the box to record the outcome of each roll (Figure 1C). This allowed us to know whether a participant had misreported their result in each trial.

The experimental procedure for participants in the individual condition (Figure 2B) was identical to that in the collaborative condition, except that the participants were told to play the game on their own. Specifically, participants were told to roll the die twice in each trial and were told that if the same number was reported twice, they would receive a reward.

There were 45 trials of dice rolling in total. The participants received a break after every 15 trials. The entire experiment lasted about 30 min. Before the experiment formally began, the participants were allowed to three practice trials. Each participant was also paid ¥15 for showing up to the experiment.

## Results

### Frequency of Lying

**Figure 3:**
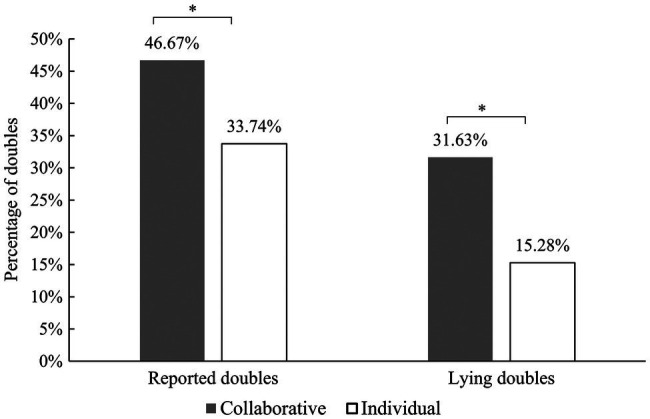
The percentage of reported doubles and the percentage of reports that were lies in both conditions. ^*^p < 0.05.

On average, participants in the collaborative condition reported 21 doubles (46.67%), and participants in the individual condition reported 15.18 doubles (33.74%). We also calculated the number of doubles participants reported by lying. As shown in Figure 3, the participants in the collaborative condition lied about 14.23 doubles (31.63%) on average, which was significantly more than the participants in the individual condition, who lied about 6.88 doubles (15.28%; Mann–Whitney U test: *U*_lying_ = 276.50, *p =* 0.027, and effect size *r* = 0.317).

**Figure 4:**
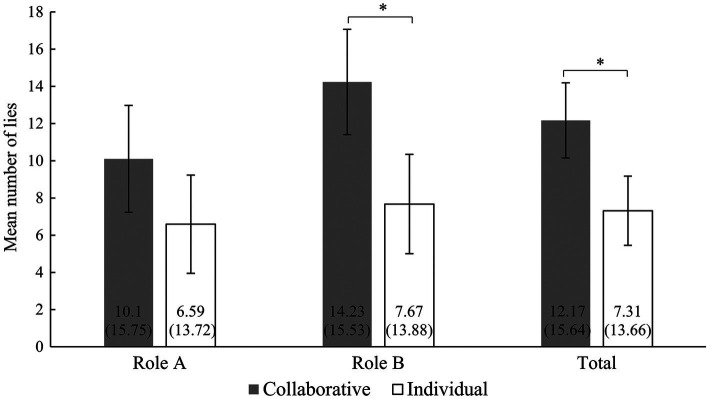
The mean number of lies for different roles in the two conditions. Error bars are ±1 SE; mean and SD are at the bottom of each bar; significance indicators: ^*^p < 0.05.

We also examined whether both A and B lied more in the collaborative condition than the participants in the individual condition. Results showed that B lied 14.23 times (*SD* = 15.53) in the collaborative condition. This means that they lied significantly more frequently than the participants did in the individual condition when playing role B (*M* = 7.67; *SD* = 13.88), *U* = 281.50, *p* = 0.034, and *r* = 0.305. In the collaborative condition, A lied 10.1 times (*SD* = 15.75), but there was no significant difference in the frequency at which A lied in the collaborative condition compared with the A in the individual condition (*M* = 6.59; *SD* = 13.72), *U* = 345.50, *p* = 0.257, and *r* = 0.112 (see Figure 4). The reports for the first and second dice rolls in the individual condition were labeled as role A and role B in the figures.

### The Number of People Who Lied

**Figure 5:**
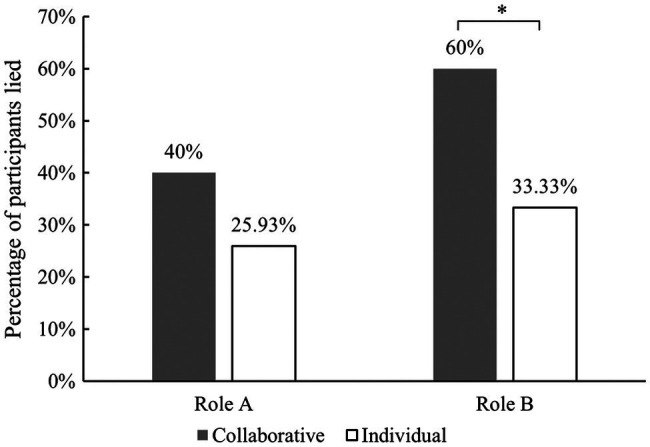
The percentage of dishonest participants in both conditions. ^*^p < 0.05.

We also calculated the number of people who lied in each condition. In the collaborative condition, 12 people playing as A (40%) lied, and 18 people playing as B (60%) lied. In the individual condition, there were seven participants (25.93%) who lied about their first roll and 9 (33.33%) who lied about their second roll (Figure 5). Cross-Tabs analysis showed that, when playing as B, more participants lied when collaborating than when operating individually (*χ^2^*(1) = 4.05, *p =* 0.044, and effect size ⱷ = 0.267). However, there was no significant difference between the number of participants who lied when playing as A in the collaborative condition compared to those who lied on their first roll in the individual condition (*χ^2^*(1) = 1.267, *p =* 0.260, and ⱷ = 0.149).

### Were Participants More Likely to Lie as the Game Progressed?

**Figure 6:**
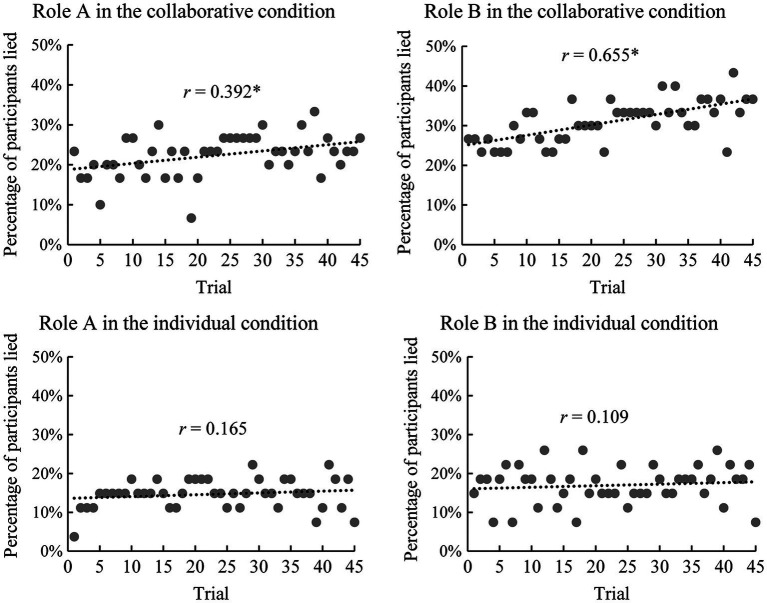
Scatter plots of the percentage of participants who lied in each trial. ^*^p < 0.05.

To investigate whether participants lied more as the game progressed, Pearson correlation analysis was used to analyze the correlation between the 45 trials and the number of participants (A and B) who lied in each trial. The results showed that the number of participants who lied increased in the collaborative condition for both A and B (*r_A_* = 0.392, *p* = 0.008; *r_B_* = 0.655, *p* < 0.001). By contrast, there were no significant correlations in the individual condition (*r_A_* = 0.165, *p* = 0.286; *r_B_* = 0.109, *p* = 0.477; see Figure 6).

## Discussion

The present study examined whether people are more dishonest in collaborative settings where there are concerns about reputation. It also examined how people’s dishonesty unfolds as they continue to collaborate. The results showed that participants told more lies in a collaborative setting than an individual setting, even though they had more reason to be concerned about their reputation in the collaborative setting. Results also showed that more participants become dishonest as they continued to collaborate.

Our results are consistent with the previous findings that indicate that participants tend to lie more in collaborative settings than in individual settings (Weisel and Shalvi, 2015; Wouda et al., 2017). Our results also show that this increased effect exists even in a face-to-face situation where there are concerns about reputation, which we introduced by asking participants to sit opposite each other at a table in this study. These findings together demonstrate that collaborative settings do indeed increase the likelihood of dishonest behavior. However, it should be noted that the participants in our study did not lie as much as the participants in the study of Weisel and Shalvi. One possible reason for this is that Weisel and Shalvi (2015) overestimated the size of the effect because the sizes of effects tend to be greater in pioneering studies that are the first to report them (Wouda et al., 2017). Another possible reason is that the participants’ concerns about their reputation in our study discouraged their dishonest behavior to a certain extent (Koch and Schmidt, 2010; Kimbrough and Rubin, 2015; Behnk et al., 2019).

Furthermore, our results for the collaborative condition showed that while B lied more than the individual participants on their second roll, A lied as frequently as the individual participants on their first roll. This finding suggests that collaboration only makes participants more dishonest when they can determine in advance whether they will be rewarded for lying. One possible reason is that participants playing as A were able to exploit the moral wiggle room provided by their partners, taking advantage of their partners’ lies without feeling morally culpable (Gross et al., 2018).

We also found that more and more participants (both A and B) lied as the game progressed. This result suggests that more participants become dishonest after they cooperate for longer. As Gachter and Falk (2002) argue, repetitive play can increase reciprocal collaboration because it is an appropriate device for re-enforcing contact. Therefore, A and B may learn to cooperate more as the task progresses, resulting in more dishonesty. Furthermore, lying in the collaborative condition benefits not just one participant but both participants, and the previous studies have revealed that prosocial lies promote trust (Levine and Schweitzer, 2015). Also, studies have shown that people who work with the same partner over time are more likely to take bribes from them as they come to trust them more (Abbink, 2004). Therefore, as participants’ interactions increase over time, they learn to trust each other more, causing them to lie more frequently when they collaborate. This finding has important implications for attempts to reduce dishonest behavior in collaborative situations. For example, Abbink (2004) suggests that rotating the players in two-player bribery games significantly reduces the amount of bribery. In socio-political spheres, many countries engage in regular staff rotation in public administration as a precautionary measure against corruption. This is the case with the Chinese civil service and the German federal government. Thus, it is reasonable to assume that it would be possible to reduce dishonest behavior in collaborative situations further by increasing staff rotation. Further research would be needed to test this hypothesis.

There are several limitations to the present study. First, the present findings suggest that individuals are more dishonest in face-to-face collaborative situations than in face-to-face individual situations. However, it should be noted that our findings may be due to the interaction between the face-to-face setting and the collaborative situation. Further studies should also include two conditions in which participants work collaboratively or individually but do not see each other’s face. This would help to examine the effect of a possible interaction between working face-to-face and working in collaboration. Second, our study indicates that individuals may not be as dishonest as Weisel and Shalvi (2015) found. This finding should be interpreted carefully because the two studies were conducted in different countries, years apart, and with different subject pools. Third, there were very few male participants, which may limit the external validity of this study. Future studies should include more male participants to examine the gender effect of collaborative dishonesty.

## Conclusion

The present study examined whether collaborative situations make individuals more dishonest in face-to-face settings and how this dishonesty unfolds over time. It found that participants whose decisions determine the final payoff (in other words, those playing as B) lied more in collaborative situations than in individual situations. Those participants whose decisions did not determine the final payoff (those playing as A) lied equally in collaborative and individual situations. Furthermore, we found that more and more participants lied as they collaborated more with their partner. These findings suggest that in face-to-face settings, collaborative situations lead to more dishonesty than individual situations.

## Data Availability Statement

The raw data supporting the conclusions of this article will be made available by the authors, without undue reservation.

## Ethics Statement

The studies involving human participants were reviewed and approved by the Ethics Committee of the Center for Cognition and Brain Disorder at Hangzhou Normal University. The patients/participants provided their written informed consent to participate in this study.

## Author Contributions

YD searched the literature, recruited the subjects, collected the data, performed the data analysis, and wrote the original draft. WM contributed to the experimental design and revised the article. QS revised the article. LS generated the research concept, provided funds, and revised the article. All authors contributed to the article and approved the submitted version.

### Conflict of Interest

The authors declare that the research was conducted in the absence of any commercial or financial relationships that could be construed as a potential conflict of interest.
